# Supplementary material for: Oleacein and Foam Cell Formation in Human Monocyte-Derived Macrophages: A Potential Strategy against Early and Advanced Atherosclerotic Lesions
Source: Pharmaceuticals (Basel). 2020 Apr 9;13(4):64. doi: 10.3390/ph13040064 (PMC7243116; doi:10.3390/ph13040064)

Supplementary Materials for

Oleacein and foam cell formation in human monocyte-derived macrophages: A potential strategy against early and advanced atherosclerotic lesions.

Filipek A.[a*], Mikołajczyk T.P.[b,c] Guzik T.J.[c,d], Naruszewicz M.[a]

[a] Department of Pharmacognosy and Molecular Basis of Phytotherapy, Faculty of Pharmacy. Medical University of Warsaw, Banacha 1, 02-097 Warsaw, Poland

[b] Institute of Infection, Immunity and Inflammation, University of Glasgow, Sir Graeme Davies Building 120 University Place, Glasgow G12 8TA, UK

[c] Department of Internal and Agricultural Medicine, Jagiellonian University Medical College, 31-007 Krakow, Poland

[d] Institute of Cardiovascular and Medical Sciences, University of Glasgow, BHF Centre for Excellence, 120 University Place, Glasgow G12 8TA, UK

* Corresponding author: Department of Pharmacognosy and Molecular Basis of Phytotherapy, Faculty of Pharmacy, Medical University of Warsaw, Banacha 1, 02-097 Warsaw, Poland

Tel./fax: +48 22 572 09 85

e-mail address: afilipek@wum.edu.pl (A.Filipek)

ORCID - Agnieszka Filipek: 0000-0002-1351-3687

Document S1

Plant material:

Ligustrum vulgare L. leaves were collected in July 2013 in Warsaw (Department of Vegetable and Medicinal Plants, Warsaw University of Life Science). Leaves were dried in the shade at room temperature. A specimen (No. LV062012) of leaves is available from the plant collection in the Department of Pharmacognosy and Molecular Basis of Phytotherapy, Medical University of Warsaw**.** The plant material was identified by Anna Kiss (Medical University of Warsaw).

Oleacein:

UHPLC-DAD-MS^n chromatograms and ^{1}H and ^{13}C NMR spectra for oleacein.

Experimental conditions:

- Oleacein was dissolved in methanol
- HPLC-DAD-MS/MS analysis was performed using analysis was performed using UHPLC-3000 RS system (Dionex, Germany) with DAD detection and an AmaZon SL mass spectrometer with ESI interface (Bruker Daltonik GmbH, Germany). Separation was performed on a Zorbax SB C18 column (150 x 2.1 mm, 1.9 mm) (Agilent, USA).
- Mobile phase A: acetonitrile/formic acid (95:5:0.01; v/v/v).
- Mobile phase B: methanol
- Linear gradient system: 0'-0%B; 60'-60%B.
- Flow rate 0.2 ml/min, column temperature 25°C.
- LC eluate was introduced into the ESI interface, without splitting, and compounds were analyzed in negative ion mode with the following settings: nebulizer pressure 40 psi; drying gas flow rate 9 l/min; nitrogen gas temperature 300C; capillary voltage 4.5 kV. The mass scan ranged from 100 to 2200 m/z. UV Spectra were recorded in the range of 200–400 nm.

- NMR experiments were recorded on a Varian VNMRS 300 (resonance frequencies 299.998 MHz for ^{1}H and 75.015 MHz for ^{13}C). Oleacein was dissolved in DMSO-d6 or CD3OD.

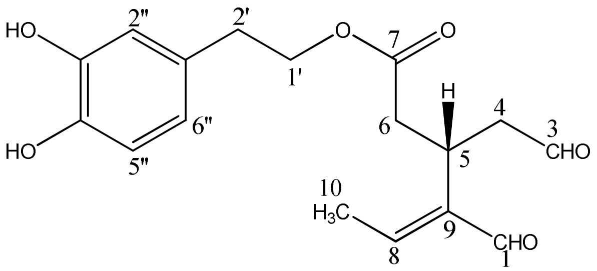

^{1}H NMR (300 MHz, $CDCl_3$): δ = 9.65 (s, 1H, H-3); 9.21 (d, 1H, *J* = 1.9 Hz, H-1); 6.79 (d, 1H, *J* = 8.3 Hz, H-5''); 6.72 (d, 1H, *J* = 2.0 Hz, H-2''); 6.66 (d, 1H, *J* = 7.31 Hz, H-8); 6.61 (dd, 1H, *J* = 8.1 Hz, 2.0 Hz, H-6''); 4.20 (m, 2H, complex, H-1'); 3.65 (m, 1H, complex, H-5); 2.81 (d, 7H, H-4); 2.06 (d, 3H, *J* = 7.1 Hz, H-10)
^{13}C NMR (75 MHz, $CDCl_3$): δ_C = 201.17 (C-3); 196.00 (C-1); 172.15 (C-7); 155.46 (C-8); 143.63 (C-4''); 143.34 (C-3''); 142.93 (C-9); 130.71 (C-1''); 121.46 (C-6''); 116.3 (C-2''); 115.45 (C-5''); 65.42 (C-1'); 46.44 (C-4); 37.16 (C-6); 34.43 (C-2'); 27.38 (C-5); 15.51 (C-10)
ESI-MS (negative): m/z 319.3

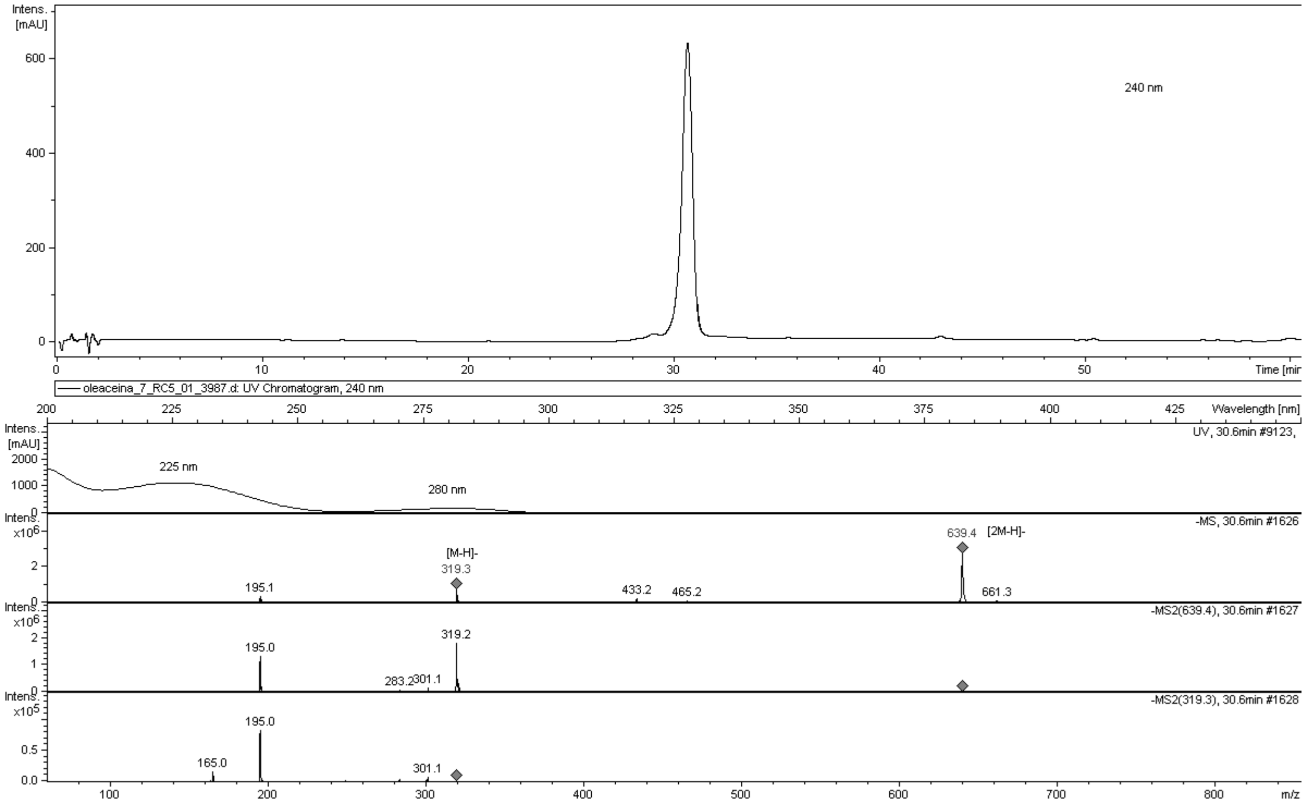

^{1}H NMR spectrum

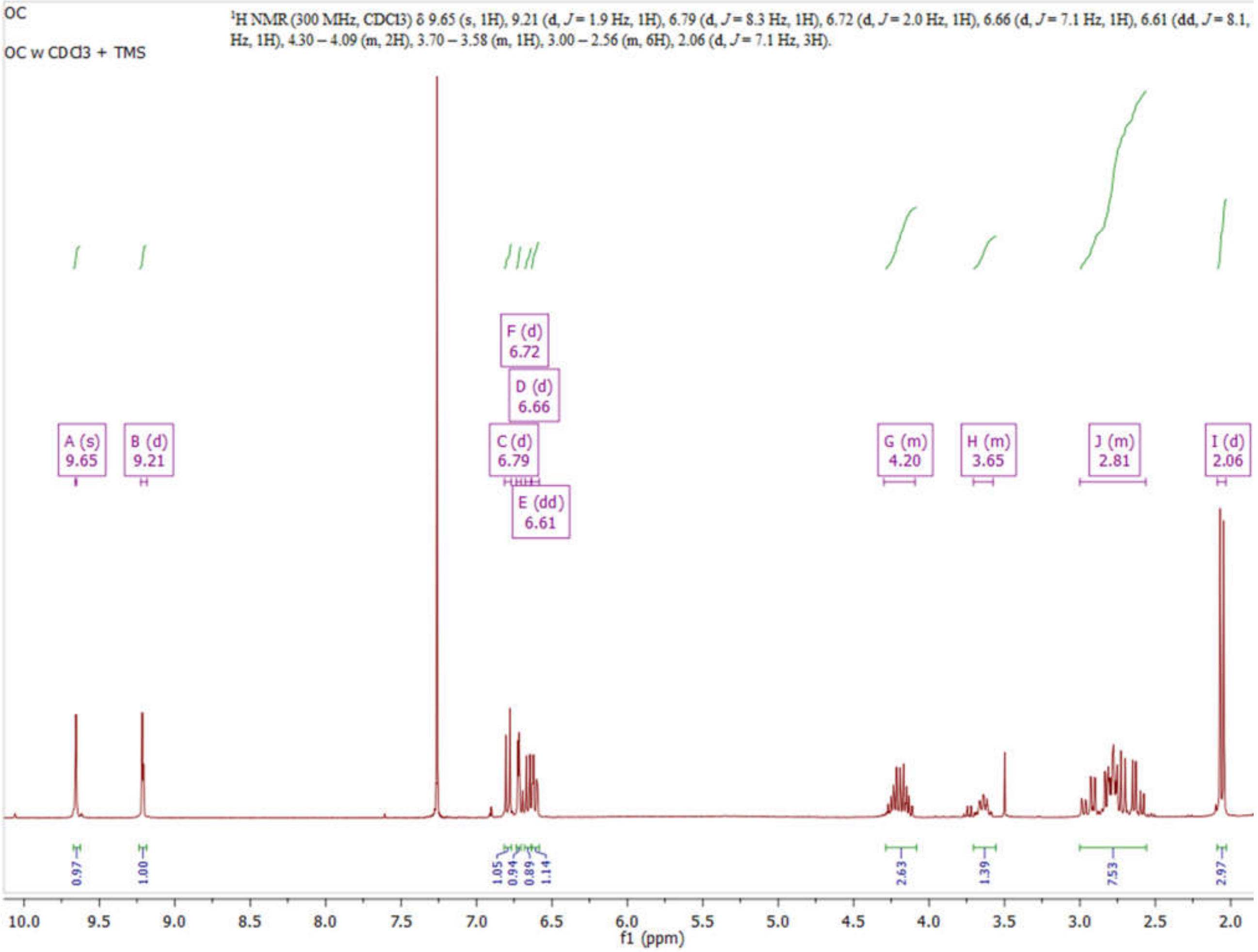

OC
OC w CDCl3 + TMS
^{1}H NMR (300 MHz, CDCl3) δ 9.65 (s, 1H), 9.21 (d, J = 1.9 Hz, 1H), 6.79 (d, J = 8.3 Hz, 1H), 6.72 (d, J = 2.0 Hz, 1H), 6.66 (d, J = 7.1 Hz, 1H), 6.61 (dd, J = 8.1,
Hz, 1H), 4.30 – 4.09 (m, 2H), 3.70 – 3.58 (m, 1H), 3.00 – 2.56 (m, 6H), 2.06 (d, J = 7.1 Hz, 3H).
A (s) 9.65
B (d) 9.21
F (d) 6.72
D (d) 6.66
C (d) 6.79
E (dd) 6.61
G (m) 4.20
H (m) 3.65
J (m) 2.81
I (d) 2.06
0.97
1.00
1.05
0.94
0.89
1.14
2.63
1.39
7.53
2.97
10.0
9.5
9.0
8.5
8.0
7.5
7.0
6.5
6.0
5.5
5.0
4.5
4.0
3.5
3.0
2.5
2.0
f1 (ppm)

^{13}C NMR spectrum

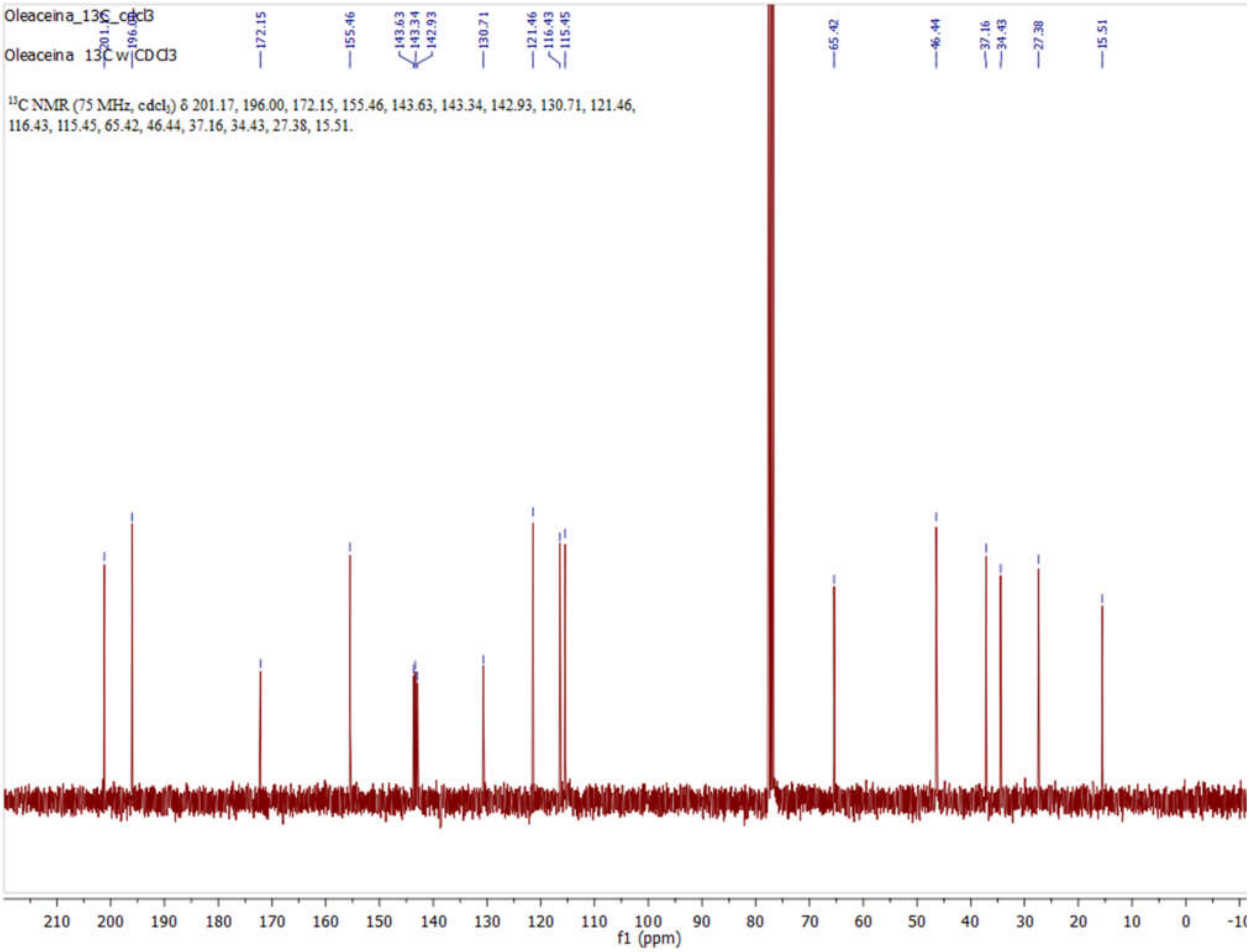

Document S2

Cytotoxicity [%] of oleacein (20 μM, 50 μM) and oxLDL (50 μg/ml, 100 μg/ml), as well as pitavastatin (20 μM) and oxLDL (50 μg/ml, 100 μg/ml) on human macrophages. Data from 3 experiments ± SEM.

oxLDL - macrophages incubated with oxLDL (50 μg/ml or 100 μg/ml); OC20, OC20+oxLDL - macrophages incubated without/with oleacein (20 μM) and oxLDL (50 μg/ml); OC50, OC50+oxLDL - macrophages incubated without/with oleacein (50 μM) and oxLDL (50 μg/ml); PIT, PIT+oxLDL - macrophages incubated without/with pitavastatin (20 μM) and oxLDL (50 μg/ml).

A

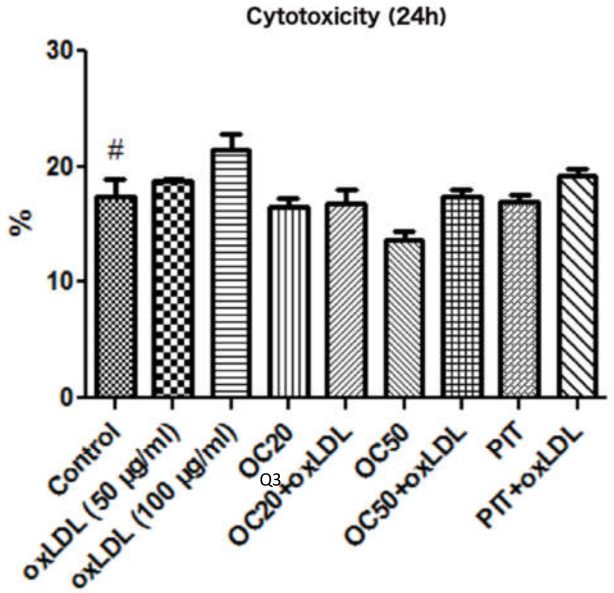

B

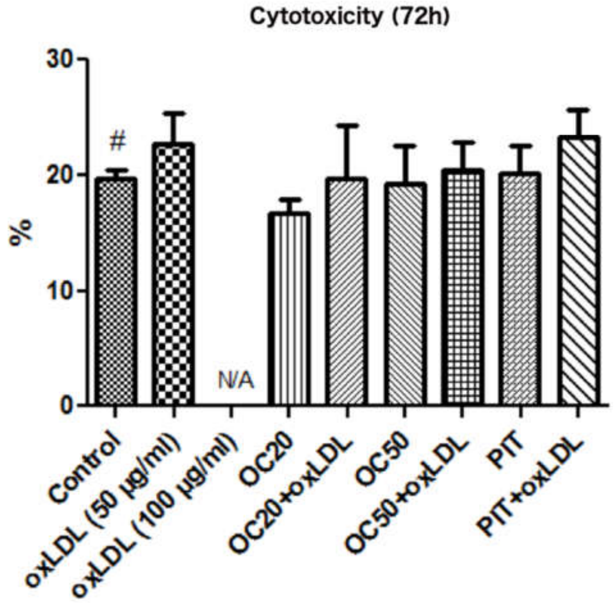

Document S3

Influence of oleacein (20 µM, 50 µM) as well as pitavastatin (20 µM) on ACAT1 concentration by oxLDL-induced macrophages was analysed by ELISA test. Data from 3 experiments ± SEM.

oxLDL - macrophages incubated with oxLDL (50 μg/ml); OC20+oxLDL - macrophages incubated with oleacein (20 μM) and oxLDL (50 μg/ml); OC50+oxLDL - macrophages incubated with oleacein (50 μM) and oxLDL (50 μg/ml); PIT+oxLDL - macrophages incubated with pitavastatin (20 μM) and oxLDL (50 μg/ml).

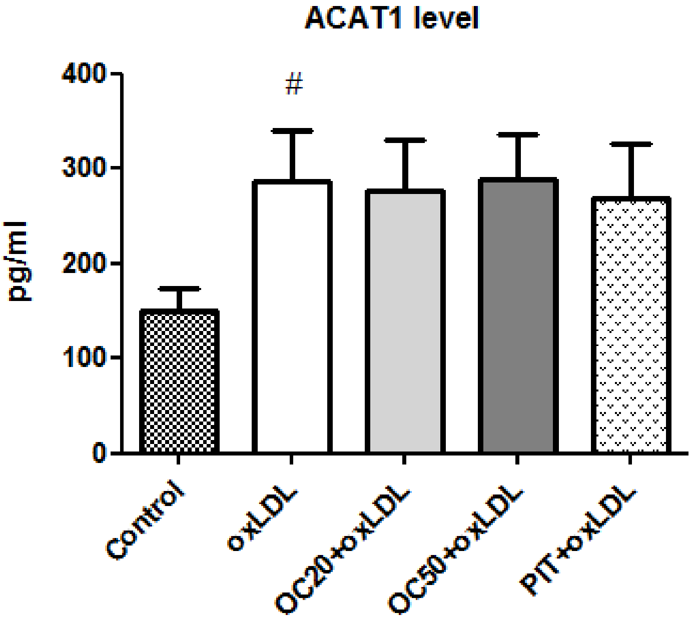

Supplement: Supplementary file 1 [file pharmaceuticals-13-00064-s001.pdf]
